# Perceived parental attitudes and gambling behavior in adults: the moderating role of resilience

**DOI:** 10.3389/fpsyg.2026.1913942

**Published:** 2026-08-12

**Authors:** Tuğba Pehlivan Sarıbudak, Arzu Dikici

**Affiliations:** 1 Department of Nursing, Faculty of Health Sciences, Acibadem Mehmet Ali Aydinlar University, Istanbul, Türkiye; 2 Nursing Department, Faculty of Health Sciences, Istanbul Arel University, Istanbul, Türkiye

**Keywords:** adults, gambling behavior, moderation analysis, parental attitudes, psychiatric nursing, resilience

## Abstract

**Introduction:**

Although multiple risk factors for problematic gambling have been identified, evidence regarding perceived parental attitudes and resilience remains limited, particularly among adults in Türkiye. This study investigated the associations between perceived parental attitudes and gambling behavior and the moderating role of resilience.

**Methods:**

A cross-sectional online correlational study was conducted in İstanbul, Türkiye, between October 2024 and May 2025 with 389 participants recruited via convenience sampling. Data were collected using the Abbreviated Perceived Parental Attitudes–Child Form, the Brief Psychological Resilience Scale, and the South Oaks Gambling Screen–Turkish Version. Moderator analyses with bias-corrected bootstrap confidence intervals were performed.

**Results:**

Maternal rejection was positively associated with gambling severity (B = 0.106, 95% CI [0.053, 0.160], *p* < 0.001), as was paternal rejection (B = 0.123, 95% CI [0.073, 0.174], *p* < 0.001). Resilience significantly moderated both associations (maternal rejection × resilience: B = −0.017, 95% CI [−0.028, −0.005], *p* = 0.005; paternal rejection × resilience: B = −0.016, 95% CI [−0.028, −0.003], *p* = 0.012). Simple slope analyses showed that the associations between parental rejection and gambling severity were significant at low (maternal: B = 0.176, *p* < 0.001; paternal: B = 0.189, *p* < 0.001) and average (maternal: B = 0.106, *p* < 0.001; paternal: B = 0.123, *p* < 0.001) levels of resilience but were not significant at high resilience (maternal: B = 0.037, *p* = 0.265; paternal: B = 0.058, *p* = 0.092). Exploratory correlation analyses additionally showed that parental emotional warmth was positively associated with resilience.

**Discussion:**

These findings suggest that resilience may buffer the association between perceived parental rejection and gambling severity.

## Introduction

Gambling has been a part of human society since ancient times and is now an everyday activity worldwide. In many countries, it is regarded as a socially acceptable form of leisure, particularly among adult populations (Ghinassi and Casale, 2023). Nevertheless, the past few years have witnessed a notable rise in gambling, which has raised concerns about its detrimental effects on individuals’ well-being and quality of life (Zhang et al., 2023; Emond et al., 2022).

Historically, the concept of “addiction” was applied primarily to psychoactive substances such as alcohol, tobacco, opioids, and cannabis. However, the DSM-5 broadened this definition to encompass behavioral addictions as well [American Psychiatric Association (APA), 2013]. Within this framework, gambling disorder is classified under the “Substance-Related and Addictive Disorders” category, alongside substance use disorders. It is defined by persistent, uncontrollable gambling behavior that continues despite significant personal, social, or occupational consequences [American Psychiatric Association (APA), 2013]. A global meta-analysis reported that the past-year prevalence of problem gambling in general adult populations ranges from 0.12 to 5.8% (Calado and Griffiths, 2016). In Türkiye, research on gambling addiction is limited, yet available data are concerning. A 2019 nationwide study involving 24,494 participants reported that 9% of participants were classified as being at high risk for gambling addiction, with this rate rising to 19.1% among single male participants aged 18–23 with a high school education (Ünübol and Hızlı Sayar, 2019). Moreover, Kovan (2024) highlighted a significant lack of evidence-based, psychotherapeutic intervention studies specifically designed for gambling addiction in Türkiye, underscoring the urgent need for further research in this area. These data highlight gambling addiction as an emerging public health concern in Türkiye.

Gambling behavior, a common form of behavioral addiction, can adversely affect individuals’ financial, social, and psychological well-being. Gambling disorder is associated with adverse outcomes such as impaired psychological functioning and reduced quality of life, as well as broader societal effects, including higher incarceration rates (Tang et al., 2020). Global research further demonstrates that gambling behavior contributes not only to psychological issues such as anxiety, depression, and stress at the individual level but also to serious social and economic consequences at the family and community levels (Kotter et al., 2019; Armoon et al., 2023). Recent evidence suggests that gambling behavior is shaped by the interaction of multiple biological, psychological, familial, and social factors rather than by a single determinant. Recent systematic reviews have identified adverse childhood experiences, maladaptive parenting, family dysfunction, psychological distress, impulsivity, financial difficulties, and substance use as major risk factors for gambling disorder. In contrast, resilience, positive family relationships, social support, and adaptive coping strategies have consistently been recognized as protective factors that may reduce gambling-related harm. These findings support a biopsychosocial perspective, emphasizing that early family experiences and individual psychological resources jointly influence vulnerability to gambling problems. Accordingly, examining perceived parental attitudes as a familial factor and resilience as an individual protective resource within the same theoretical framework may provide a more comprehensive understanding of gambling behavior (Moreira et al., 2023; Ghelfi et al., 2024).

Parental attitudes play a key role in shaping individuals’ emotional, cognitive, and social development and have been linked to gambling behaviors. Overprotective, neglectful, or rejecting parenting is associated with low self-esteem, feelings of inadequacy, and heightened anxiety in children, which may increase susceptibility to risky behaviors, including gambling, later in life (Kaya and Deveci, 2022). Recent reviews indicate that maladaptive parenting, poor family functioning, limited parental support, and adverse childhood experiences are consistently associated with an increased risk of gambling-related problems, whereas supportive family relationships appear to reduce gambling-related harm (Moreira et al., 2023; Smith et al., 2026). Weak parent–child relationships (Serna et al., 2023), insufficient family social support (Riley et al., 2021), and low levels of communication and trust with parents (Kaya and Deveci, 2022) have also been associated with increased gambling behaviors. Conversely, positive parent–child interactions can mitigate gambling engagement among adolescents (Riley et al., 2021). Research on substance use disorders indicates that individuals often perceive their parents as rejecting, aggressive, or neglectful (Moreira et al., 2023; Arslan, 2022). While parental discipline may protect against low-risk gambling, it does not prevent high-risk gambling among adolescents (Serna et al., 2023). Studies on internet gaming disorder further suggest that parent–child conflict and family disharmony elevate the risk of addictive behaviors, whereas positive parenting and parental knowledge have protective effects (Petrescu et al., 2025). Taken together, these findings underscore the potential impact of perceived parental acceptance and rejection on both the developmental trajectory and psychological adjustment of individuals who gamble. Taken together, these findings underscore the potential influence of perceived parental acceptance and rejection on the developmental trajectory and psychological adjustment of individuals who gamble. However, despite increasing interest in familial determinants of gambling behavior, empirical studies specifically examining the association between perceived parental attitudes and gambling behavior in adults remain limited (Moreira et al., 2023; Ghelfi et al., 2024). Therefore, perceived parental attitudes may be conceptualized as important familial risk and protective factors that shape vulnerability to gambling behavior across the lifespan.

Resilience refers to an individual’s capacity to adapt flexibly and recover from adverse life events and stress, emerging stronger from challenges (Padmanabhanunni et al., 2023). Research indicates that resilience not only buffers the impact of stress but also supports emotional regulation, effective problem-solving, and adaptive decision-making, all of which mitigate engagement in addictive behaviors (Padmanabhanunni et al., 2023). Individuals with high resilience are better able to employ adaptive coping strategies in the face of stress and negative experiences, reducing their vulnerability to risky behaviors such as problem gambling (Bush et al., 2021; Sirola et al., 2023). More specifically, in the context of gambling behavior, resilience functions as a protective factor, with individuals who have higher resilience being less likely to develop gambling problems even when exposed to environmental or familial risks (Sirola et al., 2023). Conversely, low resilience is associated with increased susceptibility to gambling-related harms, including financial, social, and psychological consequences, particularly among those with adverse childhood experiences or maladaptive parental relationships (Moreira et al., 2023). While Mishra et al. (2019) examined resilience-related components—including self-efficacy, mindfulness, and grit—as protective factors explaining variance in problem gambling tendencies, Cohen and Jones (2022) showed that psychological resilience and family dysfunction functioned as mediators in the relationship between parental behavioural addictions and adult mental health outcomes. Together, these findings suggest that resilience operates as a protective resource across different contexts by reducing the impact of both gambling-related and familial risk factors. Accordingly, resilience may play an important role in the relationship between perceived parental attitudes and gambling behaviors. Although rejecting or overly controlling parental behaviors may increase vulnerability to gambling and other addictive tendencies, resilience can buffer these adverse influences and reduce the likelihood of problematic gambling. This interpretation is consistent with broader evidence highlighting the central role of healthy family functioning in fostering resilience. Examining resilience as a moderator therefore helps explain how early familial experiences shape adult gambling behaviors and identifies potential targets for prevention. Mishra et al. (2019) also emphasized the importance of strengthening modifiable protective factors, such as self-efficacy and mindfulness, in prevention efforts. Taken together, these findings suggest that resilience functions as a protective psychological resource capable of buffering the adverse influence of maladaptive parental attitudes on gambling behavior. Examining resilience as a moderator may therefore help explain why individuals exposed to similar familial risk factors differ in their susceptibility to gambling-related problems (Ghelfi et al., 2024).

It has been suggested that factors influencing gambling behavior should be investigated in populations with larger sample sizes and diverse cultural characteristics (Zhang et al., 2023). Studies directly examining the relationship between parental attitudes, resilience, and gambling behavior remain limited. While various risk factors for problematic gambling behavior have been identified, relatively less attention has been given to understanding protective factors that could reduce risk or buffer against the development of problematic gambling tendencies. Therefore, the general objective of this study was to examine the associations between perceived parental attitudes and gambling behavior in adults and to investigate whether resilience moderates these associations. Specifically, the study aimed to determine whether maternal and paternal rejection, overprotection, and emotional warmth were associated with gambling severity and whether these associations differed according to individuals’ levels of resilience. Based on these objectives, the hypotheses presented below were formulated. The findings of this study are expected to contribute to a better understanding of the interplay between familial and individual factors associated with gambling behavior and to provide evidence that may inform future prevention and intervention strategies.

Based on the existing theoretical and empirical evidence, the following hypotheses were proposed:

### Primary (main-effect) hypotheses

*H1a*: Higher maternal rejection (MRA) will be associated with higher gambling severity (SOGS).

*H1b*: Higher paternal rejection (PRA) will be associated with higher gambling severity (SOGS).

*H1c*: Higher maternal overprotection (MOP) will be associated with higher gambling severity (SOGS).

*H1d*: Higher paternal overprotection (POP) will be associated with higher gambling severity (SOGS).

*H1e*: Higher maternal emotional warmth (MEW) will be associated with lower gambling severity (SOGS).

*H1f*: Higher paternal emotional warmth (PEW) will be associated with lower gambling severity (SOGS).

### Moderator (buffering) hypotheses

*H2a*: Resilience will moderate the association between MRA and SOGS, such that the positive association is weaker at higher levels of resilience.

*H2b*: Resilience will moderate the association between PRA and SOGS, such that the positive association is weaker at higher levels of resilience.

*H2c*: Resilience will moderate the association between MOP and SOGS, such that the positive association is weaker at higher levels of resilience.

*H2d*: Resilience will moderate the association between POP and SOGS, such that the positive association is weaker at higher levels of resilience.

*H2e*: Resilience will moderate the association between MEW and SOGS, such that the negative association is stronger at higher levels of resilience.

*H2f*: Resilience will moderate the association between PEW and SOGS, such that the negative association is stronger at higher levels of resilience.

## Methods

### Aims

This study aimed to examine the effects of perceived parental attitudes on gambling behavior in adults and to investigate the moderating role of resilience.

### Research design

A cross-sectional correlational study was conducted using an electronic questionnaire administered online between October 2024 and May 2025. The survey link was disseminated via social media and professional networks. Online questionnaires are widely accepted in health sciences research as they facilitate efficient data collection and enhance data completeness (Ebert et al., 2018; Uhlig et al., 2014). Given the cross-sectional correlational design, the findings should be interpreted as associations rather than causal effects. The study was reported following the Strengthening the Reporting of Observational Studies in Epidemiology (STROBE) guidelines (von Elm et al., 2007).

### Setting and participants

The target population of this study comprised adults residing in Istanbul between October 2024 and May 2025. The required sample size was calculated using the OpenEpi program, based on the number of adult residents reported in the Türkiye Demographic and Health Survey (2018) data (Hacettepe University Institute of Population Studies, 2019). Assuming a known population size, a 5% margin of error, and a 95% confidence interval, the minimum sample size was determined to be 384 participants (Dean et al., 2013). Ultimately, 389 individuals who met the inclusion criteria and voluntarily agreed to participate were enrolled in the study. A convenience sampling method was employed. All submitted questionnaires were complete and met the eligibility criteria; therefore, no missing data were observed, no cases were excluded because of incomplete responses, and complete-case analysis was performed. The completion rate of the online survey was 100% among submitted questionnaires. The *post hoc* power analysis was conducted after completion of the primary analyses and indicated a statistical power of 99%, suggesting that the sample size was sufficient to detect the observed effect.

*Inclusion criteria*: voluntary consent to participate, age ≥18 years, literacy, and no vision- or hearing-related communication or comprehension impairments.

*Exclusion criteria*: presence of intellectual disability or cognitive impairment, inability to complete the questionnaires due to physical limitations, or severe psychiatric symptoms requiring treatment in an acute psychiatric unit.

### Instruments

#### Personal information form

This 21-item form, developed by the researchers based on the relevant literature, collected data on participants’ demographic characteristics (age, gender, education level, marital status), alcohol and tobacco use, history of chronic and psychiatric illness, and gambling behavior.

#### Abbreviated Perceived Parental Attitudes–Child Form

This scale measures parental attitudes experienced during childhood across three subscales: emotional warmth, overprotection, and rejection. The 23-item abbreviated version developed by Arrindell et al. (1999) was employed in this study. The Turkish adaptation was conducted by Dirik et al. (2015) using university students. In that study, the internal consistency coefficients for paternal attitudes were 0.79 for emotional warmth, 0.73 for overprotection, and 0.71 for rejection, while the coefficients for maternal attitudes were 0.75, 0.72, and 0.64, respectively. In the present study, the internal consistency coefficients (Cronbach’s *α*) were 0.71 for Maternal Overprotective Attitudes, 0.71 for Paternal Overprotective Attitudes, 0.83 for Maternal Rejecting Attitudes, 0.86 for Paternal Rejecting Attitudes, 0.86 for Maternal Emotional Warmth Attitudes, and 0.87 for Paternal Emotional Warmth Attitudes. For analysis, composite variables were generated by summing the total scores for perceived maternal and paternal attitudes in the three domains: emotional warmth, overprotection, and rejection. Because the three subscales contain different numbers of items, their raw total scores are not directly comparable and should be interpreted only within each subscale rather than across subscales.

#### Brief Resilience Scale (BRS)

This scale, developed by Smith et al. (2008), assesses individuals’ ability to recover, regain prior functioning, and adapt following adversity. Adapted into Turkish by Doğan (2015), it is a single-dimension, 6-item scale rated on a 5-point Likert scale (1 = not appropriate at all, 5 = completely appropriate). Items 2, 4, and 6 are reverse-coded. Total scores range from 6 to 30, with higher scores indicating greater resilience. The Turkish version demonstrated good reliability, with a Cronbach’s alpha of 0.79 in the adaptation study and 0.81 in the current study.

#### South Oaks Gambling Screen–Turkish Version (SOGS-TV)

The South Oaks Gambling Screen (SOGS), initially developed by Lesieur and Blume (1987), is a 26-item self-report instrument designed to assess the severity of gambling behavior. In the original version, the first three items, along with items 12, 16j, and 16 k, were excluded from scoring, yielding a total of 20 items. For the Turkish adaptation, three items identified by reliability experts were removed, and two culturally specific items were added, resulting in a 19-item version with a cutoff score of 8. Each item is scored as 1 point, with no reverse scoring, for a maximum possible score of 19 (Duvarcı and Varan, 2001). The Turkish version demonstrated strong reliability, with a Cronbach’s alpha of 0.88 in the adaptation study and 0.84 in the present study.

### Research team

All researchers are psychiatric nurses with expertise in their respective fields, including an associate professor and an assistant professor. They also have 10–11 years of experience in clinical nursing. They are active members of professional organizations, such as the Psychiatric Nurses Association, and contribute to advancements in psychiatric care.

### Data analysis

Data analysis was conducted using IBM SPSS Statistics version 29.0 and Jamovi version 2.3. Prior to creating the interaction terms, all predictor and moderator variables were mean-centered to reduce potential multicollinearity and facilitate interpretation of the interaction effects. Categorical variables were summarized as frequencies and percentages, whereas numerical variables were presented as means, standard deviations, medians, minimums, and maximums. The distribution of continuous variables was evaluated using the Kolmogorov–Smirnov and Shapiro–Wilk tests, together with skewness, kurtosis, Q–Q plots, and histogram inspection. Because the SOGS scores showed marked non-normality and a high proportion of zero values, Spearman’s rank correlation coefficients were additionally calculated as a sensitivity analysis and compared with the Pearson correlation coefficients. As the Spearman and Pearson analyses yielded highly similar correlation coefficients, directions of association, and significance patterns, the Pearson coefficients are presented to maintain consistency with the moderation analyses. Relationships between numerical variables were examined using Pearson’s correlation coefficient.

Analyses of the models regarding the moderating role of resilience were conducted. Confidence intervals for the estimates of the moderation models were obtained using the bias-corrected bootstrap method. Moderation analyses were performed using ordinary least squares (OLS) regression because the primary objective of the study was to examine interaction effects between parental attitudes and resilience. Bias-corrected bootstrap confidence intervals were used to obtain more robust estimates of the regression coefficients. The moderation analyses were performed using the MedMod module in Jamovi (version 2.3). The estimates reported from these analyses are unstandardized regression coefficients (*B*). The MedMod module provides regression coefficients, standard errors, 95% confidence intervals, significance tests, interaction effects, and conditional (simple slope) effects. However, the version of the MedMod module used in the present study does not report overall model-fit statistics (*R*^2^ and *F*), the change in explained variance attributable to the interaction term (Δ*R*^2^), or an interaction-specific effect-size index. Therefore, the moderation effects were evaluated based on the interaction coefficients, their 95% confidence intervals, *p* values, and the conditional effects estimated at the mean and ±1 SD levels of resilience. Although the SOGS scores exhibited a positively skewed and zero-inflated distribution, the OLS-based moderation approach was retained because it directly addresses the study hypothesis regarding interaction effects. Nevertheless, the distributional characteristics of the outcome variable were explicitly considered when interpreting the findings and are acknowledged as a study limitation. In moderation models where the interaction effect was found to be significant, the effects of Maternal Rejecting Attitudes and Paternal Rejecting Attitudes on SOGS were reported for individuals with different levels of resilience (Gürbüz, 2025). Statistical significance was set at *α* = 0.05.

### Ethical considerations

This study was conducted in accordance with the ethical principles outlined in the Declaration of Helsinki. Before data collection, ethical approval was granted by the İstanbul Arel University Ethics Committee (Approval No: 20, October 11, 2024). Participation was entirely voluntary, and all individuals were informed about the purpose of the research, study procedures, and their rights as participants. Written informed consent was obtained electronically from all participants prior to their participation in the study. Participants provided their consent by completing and submitting the online consent form before accessing the questionnaire. Participants were assured of their right to withdraw from the study at any time without penalty. Anonymity and confidentiality were strictly maintained; no identifying information was collected, and all data were securely stored on password-protected servers accessible only to the research team.

## Results

Table 1 summarizes the key demographic and clinical characteristics of the study sample. The participants were predominantly female (68.4%, *n* = 266), with a mean age of 29.27 years (range: 18–64). Most participants were single (65.3%), childless (72.0%), and university graduates (59.6%). More than half were employed (55.3%), and the majority resided with their families (76.9%). Regarding health status, 10.5% reported having a chronic illness, while 6.2% had a history of psychiatric disorders, most commonly anxiety and depression. Participation in gambling or games of chance was reported by 23.9% of participants, primarily for entertainment or financial gain. The vast majority (84.3%) reported spending less than 1,000 TL per month on such activities.

**Table 1:** Demographic information.

Variable	X̄±SD/*n*	Median (min–max)/%
Age	29.27 ± 10.41	25 (18–64)
Gender		
Male	123	31.6
Female	266	68.4
Marital status		
Single	254	65.3
Married	135	34.7
Having a child		
Yes	109	28.0
No	280	72.0
Number of children		
0	280	72.0
1	46	11.8
2	47	12.1
3	15	3.9
5	1	0.3
Educational status		
High school	91	23.4
Master’s/PhD	64	16.5
Bachelor’s degree	232	59.6
Primary school–literate	2	0.5
Occupation		
Student	132	33.9
Healthcare workers	101	26.0
Teacher	56	14.4
Other	33	8.5
Engineer	26	6.7
Self-employed	24	6.2
Unemployed	17	4.4
Employment status		
Yes	215	55.3
No	174	44.7
Whom living with		
Family	299	76.9
Relatives	5	1.3
Friends	10	2.6
Alone	35	9.0
Dormitory	40	10.3
Monthly income status		
Income < expenses	76	19.5
Income > expenses	77	19.8
Income = expenses	236	60.7
Having a chronic illness		
Yes	41	10.5
No	348	89.5
Mental illness history		
Yes	24	6.2
No	365	93.8
Alcohol consumption		
Yes	111	28.5
No	278	71.5
Smoking		
Yes	118	30.3
No	271	69.7
Current gambling		
Occasionally	53	13.6
Quit	32	8.2
Yes	8	2.1
Never	296	76.1
Age of first gambling (*n* = 89)		
10–15	8	9.0
16–18	32	36.0
19–22	34	38.2
>23	15	16.9
Duration of gambling (*n* = 78)		
<1 year	43	55.1
1–5 years	23	29.5
5–10 years	6	7.7
10–15 years	4	5.1
>15 years	2	2.6
Reasons for gambling*		
Entertainment	117	30.5
To earn money	71	18.5
Excitement	54	14.1
Avoidance	30	7.8
Socialization	11	2.9
Monthly gambling expenditure (*n* = 70)		
<1,000 TL	59	84.3
1,000–50,000 TL	11	15.7
Reaction to frequent losses		
Totally disagree	328	84.3
Partially agree	39	10.0
Totally agree	22	5.7

X̄, arithmetic mean; SD, standard deviation; Min, minimum; Max, maximum. Participants could select more than one response; therefore, percentages may not sum to 100%. Percentages were calculated using the number of participants who answered this item as the denominator.

Descriptive statistics for the study variables and their intercorrelations are presented in Table 2. Strong positive correlations were observed between the corresponding maternal and paternal parental-attitude subscales: maternal and paternal overprotection (*r* = 0.73, *p* < 0.001), maternal and paternal rejection (*r* = 0.61, *p* < 0.001), and maternal and paternal emotional warmth (*r* = 0.76, *p* < 0.001). However, these variables were examined in separate moderation models and were therefore not entered simultaneously as predictors. The correlation between resilience and SOGS was negligible and nonsignificant (*r* = −0.01, *p* = 0.80), indicating that resilience was not directly associated with gambling severity in the present sample, despite its moderating role in the rejection models. Pearson’s correlation coefficients are reported as the primary analyses, whereas Spearman’s rank correlations were conducted as sensitivity analyses. The mean resilience score was 19.56 ± 4.20 (range: 6–30). Maternal overprotection averaged 20.11 ± 5.00 (10–36), paternal overprotection 18.99 ± 4.99 (9–36), maternal rejection 10.14 ± 3.36 (7–25), and paternal rejection 9.84 ± 3.49 (7–27). Maternal emotional warmth was 19.25 ± 4.91 (7–28), and paternal emotional warmth was 18.28 ± 5.24 (7–28). The mean South Oaks Gambling Screen (SOGS) score was 0.75 ± 1.82, with values ranging from 0 to 10. The SOGS distribution was markedly zero-inflated and positively skewed (median = 0, skewness = 3.24, kurtosis = 11.02). Normality tests confirmed a significant deviation from normality (Kolmogorov–Smirnov = 0.411, *p* < 0.001; Shapiro–Wilk = 0.476, *p* < 0.001). Overall, 292 participants (75.1%) obtained a SOGS score of zero, confirming the highly zero-inflated nature of the outcome variable. Spearman’s rank correlation analyses yielded findings that were highly consistent with the Pearson correlations, with no meaningful differences in the direction or statistical significance of the associations. In particular, maternal rejection (*ρ* = 0.15, *p* < 0.01) and paternal rejection (*ρ* = 0.22, *p* < 0.001) remained positively associated with SOGS scores. These findings indicate that the observed associations were robust across both parametric and non-parametric correlation analyses despite the non-normal distribution of the outcome variable.

**Table 2:** Descriptive statistics and Pearson correlations among study variables (*n* = 389).

Variable	*M*	*SD*	Median	Min	Max	1	2	3	4	5	6	7
1. Resilience	19.56	4.20	19	6	30	—						
2. Maternal overprotection	20.11	5.00	19	10	36	0.02	—					
3. Paternal overprotection	18.99	4.99	19	9	36	−0.05	0.73*	—				
4. Maternal rejection	10.14	3.36	9	7	25	−0.03	0.52*	0.27*	—			
5. Paternal rejection	9.84	3.49	9	7	27	−0.07	0.27*	0.43*	0.61*	—		
6. Maternal emotional warmth	19.25	4.91	20	7	28	0.15**	0.01	0.06	−0.43*	−0.26*	—	
7. Paternal emotional warmth	18.28	5.24	19	7	28	0.14**	0.04	0.08	−0.31*	−0.39*	0.76*	—
8. SOGS	0.75	1.82	0	0	10	−0.01	−0.01	0.02	0.17**	0.22*	−0.06	−0.07

M, mean; SD, standard deviation; Min, minimum; Max, maximum; SOGS, South Oaks Gambling Screen. Values are Pearson correlation coefficients. *p* < 0.05, **p < 0.01, ***p < 0.001. Because no correlations reached the 0.05 level without also reaching the 0.01 level, no single-asterisk coefficients appear in the table. The three parental-attitude dimensions were examined in separate moderation models; therefore, the high correlations between corresponding maternal and paternal subscales were not entered simultaneously into the same model.

The results of the model regarding the moderating role of resilience in the relationship between parental attitudes and gambling behavior are presented in Table 3. Six different models were established for parental attitudes, namely maternal and paternal overprotection, maternal and paternal rejection, and maternal and paternal emotional warmth. Among these, only Model 3 and Model 4 showed statistically significant results for parental attitudes (MRA and PRA, respectively) and for the interaction effect of parental attitudes with resilience. In the other models, neither the main effects nor the interaction effects reached statistical significance (Table 3; Model 1: *p* = 0.855, 0.802, and 0.612; Model 2: *p* = 0.639, 0.840, and 0.576; Model 5: *p* = 0.263, 0.952, and 0.752; Model 6: *p* = 0.194, 0.956, and 0.883). The estimates presented in Table 3 are unstandardized regression coefficients (*B*). Accordingly, all coefficients in the text are interpreted in the original measurement units of the variables. The statistical evidence for moderation is presented through the interaction coefficients, their 95% confidence intervals, significance tests, and conditional effects estimated at the mean and ±1 SD levels of resilience. The moderation module used for the analyses did not provide overall model-fit statistics, changes in explained variance attributable to the interaction term, or an interaction-specific effect-size index. Therefore, *R*^2^, Δ*R*^2^, *F* statistics, and Cohen’s *f*^2^ could not be reported for these models.

**Table 3:** Moderation model estimates.

Predictor	*B*	SE	Lower (95% CI)	Upper (95% CI)	*Z*	*p*
Model 1						
MOPA	−0.003	0.019	−0.040	0.033	−0.183	0.855
Resilience	−0.006	0.022	−0.049	0.038	−0.251	0.802
MOPA × resilience	−0.002	0.004	−0.010	0.006	−0.507	0.612
Model 2						
POPA	0.009	0.018	−0.028	0.045	0.469	0.639
Resilience	−0.004	0.022	−0.048	0.039	−0.202	0.840
POPA × resilience	−0.002	0.004	−0.011	0.006	−0.559	0.576
Model 3						
MRA	0.106	0.027	0.053	0.160	3.871	<0.001
Resilience	−0.003	0.021	−0.045	0.039	−0.134	0.894
MRA × resilience	−0.017	0.006	−0.028	−0.005	−2.806	0.005
Conditional effects of MRA at levels of resilience						
Average	0.106	0.028	0.052	0.161	3.840	<0.001
Low (−1SD)	0.176	0.041	0.096	0.256	4.300	<0.001
High (+1SD)	0.037	0.033	−0.028	0.102	1.110	0.265
Model 4						
PRA	0.123	0.026	0.073	0.174	4.794	<0.001
Resilience	0.003	0.021	−0.039	0.045	0.145	0.885
PRA × resilience	−0.016	0.006	−0.028	−0.003	−2.510	0.012
Conditional effects of PRA at levels of resilience						
Average	0.123	0.026	0.073	0.174	4.760	<0.001
Low (−1SD)	0.189	0.039	0.112	0.265	4.850	<0.001
High (+1SD)	0.058	0.035	−0.010	0.126	1.680	0.092
Model 5						
MEW	−0.021	0.019	−0.058	0.016	−1.120	0.263
Resilience	−0.001	0.022	−0.044	0.042	−0.061	0.952
MEW × resilience	−0.001	0.005	−0.010	0.007	−0.315	0.752
Model 6						
PEW	−0.023	0.018	−0.057	0.012	−1.299	0.194
Resilience	−0.001	0.022	−0.044	0.042	−0.056	0.956
PEW × resilience	−0.001	0.004	−0.009	0.008	−0.148	0.883

The dependent variable was SOGS, and resilience was examined as the moderator. *B*, unstandardized regression coefficient; SE, standard error; CI, confidence interval. Predictor and moderator variables were mean-centered before the interaction terms were constructed. Conditional effects represent the association between the predictor and SOGS at the mean and at 1 SD below and above the mean of resilience. Conditional-effect rows are presented separately from the main model coefficients. The version of the MedMod module used did not report *R*^2^, Δ*R*^2^, overall *F* statistics, or an interaction-specific effect-size index. MOPA, maternal overprotective attitudes; POPA, paternal overprotective attitudes; MRA, maternal rejecting attitudes; PRA, paternal rejecting attitudes; MEW, maternal emotional warmth attitudes; PEW, paternal emotional warmth attitudes; SOGS, south oaks gambling screen.

**Figure 1:**
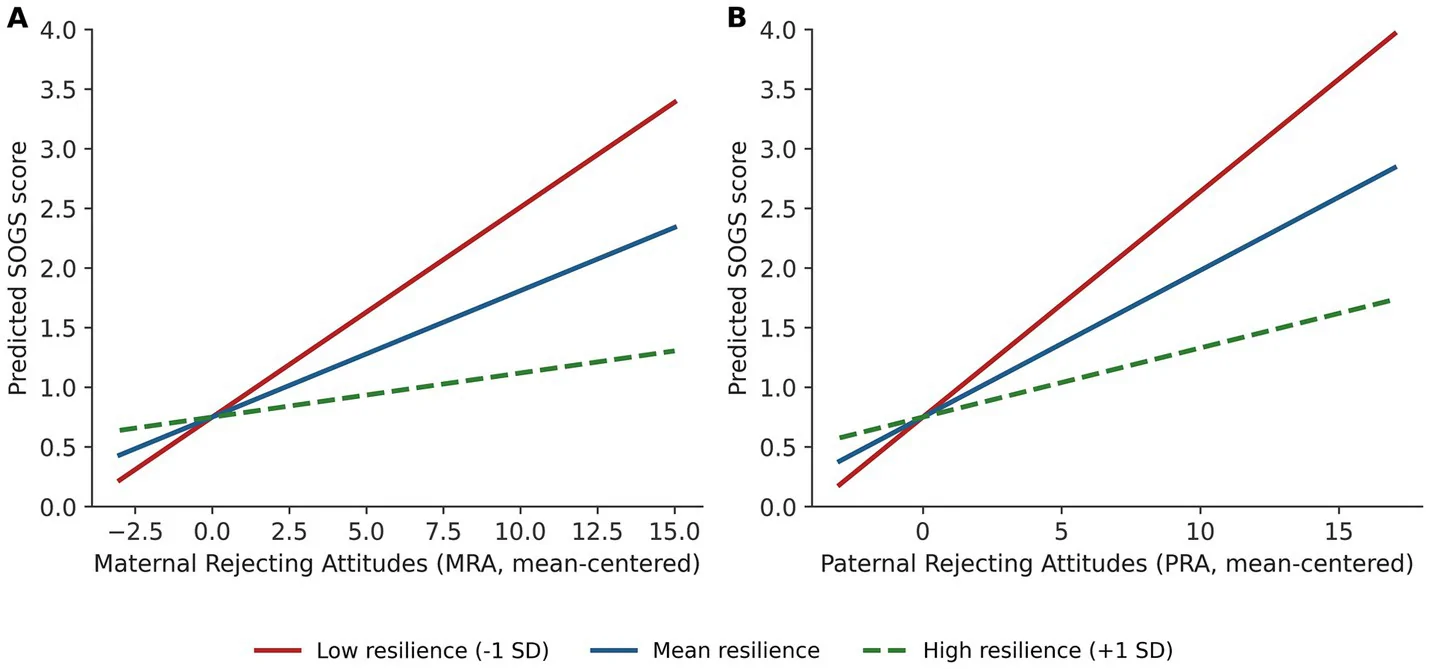
Simple slope graphs for Model 3 and Model 4. **(A)** Model 3: the association between Maternal Rejecting Attitudes (MRA) and SOGS at low, mean, and high levels of resilience. **(B)** Model 4: the association between Paternal Rejecting Attitudes (PRA) and SOGS at low, mean, and high levels of resilience. Predictor variables were mean-centered prior to constructing the interaction terms. MRA, Maternal Rejecting Attitudes (mean-centered); PRA, Paternal Rejecting Attitudes (mean-centered); SOGS, South Oaks Gambling Screen.

In Model 3, a statistically significant association was observed between maternal rejection (MRA) and SOGS scores; a one-unit increase in MRA was associated with an average increase of 0.106 units in the SOGS score (Table 3, Model 3, *B* = 0.106, *p* < 0.001). However, since the interaction term between MRA and resilience (*B* = −0.017) was also statistically significant (Table 3, Model 3, *p* = 0.005), the association between MRA and SOGS varied according to the level of resilience. The negative regression coefficient for the interaction term suggests that the association between maternal rejection and SOGS became weaker as resilience increased. The association between MRA and SOGS was further examined at different levels of resilience. As shown in Figure 1A, among individuals with average resilience or resilience 1 SD below the mean, the association between MRA and SOGS (*B* = 0.106 and *B* = 0.176, respectively) was statistically significant (*p* < 0.001). In contrast, among individuals with resilience 1 SD above the mean, this association (*B* = 0.037) was not statistically significant (*p* = 0.265). These findings suggest that the association between maternal rejection and gambling severity was stronger among individuals with lower resilience and weaker among those with higher resilience.

In Model 4, **a statistically significant association was observed between paternal rejection (PRA) and SOGS scores**; a one-unit increase in PRA was associated with an average increase of 0.123 units in the SOGS score (Table 3, Model 4, *B* = 0.123, *p* < 0.001). However, since the interaction term between PRA and resilience (*B* = −0.016) was also statistically significant (Table 3, Model 4, *p* = 0.012), **the association between PRA and SOGS varied according to the level of resilience.** The negative regression coefficient for the interaction term suggests that **the association between paternal rejection and SOGS became weaker as resilience increased. The association between PRA and SOGS** was further examined at different levels of resilience. As shown in Figure 1B, among individuals with average resilience or resilience 1 *SD* below the mean, **the association between PRA and SOGS** (*B* = 0.123 and *B* = 0.189, respectively) was statistically significant (*p* < 0.001). In contrast, among individuals with resilience 1 *SD* above the mean, this association (*B* = 0.058) was not statistically significant (*p* = 0.092), indicating that the association between paternal rejection and gambling severity was no longer statistically significant at higher levels of resilience.

## Discussion

To the best of the authors’ knowledge, this study is among the first to examine the moderating role of resilience in the relationship between parental attitudes and gambling behavior among adults. Although the findings provide novel evidence from a convenience sample of adults living in İstanbul, they should not be interpreted as representative of the broader Turkish adult population.

In the current study, participants’ low average scores on the South Oaks Gambling Screen (SOGS) indicate that problematic gambling behavior was generally low among the sample. A global meta-analysis reported that the past-year prevalence of problem gambling in general adult populations ranges from 0.12 to 5.8% (Calado and Griffiths, 2016). Studies conducted in Türkiye indicate that the prevalence of gambling disorder varies depending on sample characteristics, age groups, and socioeconomic factors. For example, Uygur (2022) reported that among 377 participants, 2.9% had severe gambling disorder and 8.2% had mild gambling disorder, totaling an 11.1% prevalence. In a nationwide study conducted in 2019 with 24,494 participants, the overall rate of high-risk gambling behavior was reported as 9%, although this rate was considerably higher (19.1%) among a specific subgroup — single, male participants aged 18–23 with a high school education (Ünübol and Hızlı Sayar, 2019). The relatively low gambling severity observed in the present sample may reflect both the community-based characteristics of the participants and sociocultural influences surrounding gambling behavior in Türkiye. Gambling continues to be socially stigmatized in Turkish society, which may discourage individuals from openly acknowledging gambling-related behaviors and may partially explain the comparatively low SOGS scores observed in this study. In Türkiye, gambling is culturally considered taboo, and individuals who gamble are often stigmatized, which may limit their willingness to report such behaviors. Moreover, low resilience or overly protective parental attitudes can increase susceptibility to risky behaviors (Zimmerman et al., 2013). In the current study, participants’ resilience levels were moderate; parental emotional warmth scores were moderate, while parental rejection scores were low, and overprotective attitudes were relatively high. When considered alongside the low SOGS scores, higher emotional warmth and lower parental rejection may reflect protective family characteristics associated with lower levels of gambling behavior. Conversely, moderate resilience levels and high overprotection scores may explain the low but existing risk of gambling. Such studies conducted with larger and more representative samples may provide more accurate estimates of gambling disorder prevalence and better inform the development of effective prevention strategies in Türkiye.

An important finding of this study is that perceived parental rejection was significantly associated with gambling behavior. A weak positive correlation was identified between SOGS scores and both maternal rejection and paternal rejection, indicating that increases in MRA and PRA were associated with higher SOGS scores. Accordingly, hypotheses H1a and H1b were supported, while the other hypotheses in this category were rejected. These findings suggest that perceived parental rejection may be associated with a greater likelihood of engaging in gambling-related behaviors. Although the observed association was relatively weak, gambling behavior is widely recognized as a multidimensional phenomenon influenced by numerous individual factors (e.g., impulsivity, emotional dysregulation, psychological distress, maladaptive coping strategies, and sensation seeking) as well as environmental factors (e.g., peer influence, family functioning, socioeconomic adversity, gambling accessibility, and cultural norms; Tran et al., 2024). Recent studies examining the link between parental rejection and risky behaviors have shown that this association varies depending on the context and type of behavior, with resilience serving as a potential buffering factor in certain situations (Kaya and Deveci, 2022). Moreover, theoretical models support the proposition that parental rejection may be related to risky behaviors by fostering maladaptive coping strategies.

According to the model results, resilience demonstrated a moderating role in specific models. While H2a and H2b were supported, the other hypotheses in this group were rejected. The findings indicate that the association between parental rejection and gambling behavior differed according to resilience levels, with higher resilience attenuating the adverse effects of both maternal and paternal rejection. Among individuals with low or average resilience, parental rejection was strongly associated with higher gambling scores, whereas for those with high resilience, this relationship was not statistically significant. These findings suggest that resilience may buffer the association between parental rejection and gambling behavior, thereby functioning as a protective factor. The literature similarly supports the moderating role of resilience in the relationship between adverse childhood experiences and psychological outcomes (Zimmerman et al., 2013; Masten, 2018). For example, Mutiso et al. (2025) reported that resilience significantly moderated the relationship between adverse childhood experiences and later psychological outcomes, including depression and post-traumatic stress symptoms, among Kenyan youth. Likewise, the present findings suggest that resilience may reduce vulnerability among individuals exposed to parental rejection. At the same time, resilience is recognized as a dynamic and culturally sensitive construct whose protective effects may vary across populations and sociocultural contexts (Liu et al., 2023). In addition, methodological characteristics, including sample size, measurement instruments, and psychometric properties, may partly explain inconsistencies across studies (Akdağ et al., 2024). Collectively, these findings support the conceptualization of resilience as a protective resource that shapes the extent to which adverse parental experiences translate into gambling-related behaviors.

An exploratory correlational finding of this study was that higher parental emotional warmth was positively associated with resilience, suggesting that supportive parenting contributes to the development of adaptive psychological resources. Previous studies consistently demonstrate that warm and supportive parenting promotes resilience and facilitates adaptive responses to stressful life events (Van Schoors et al., 2023; Masten and Tellegen, 2012). Similarly, Wang et al. (2024) found that parental emotional warmth reduced emotional and behavioral problems among adolescents, whereas parental rejection increased these problems, with resilience partially mediating the relationship between parenting style and adolescents’ emotional and behavioral problems. These findings suggest that parental rejection represents a potential familial risk factor for gambling behavior, whereas parental emotional warmth may serve as an important protective factor by fostering resilience. Nevertheless, the relatively weak associations observed in the present study indicate that gambling behavior is likely to be influenced by additional cultural, socioeconomic, familial, and individual characteristics beyond parenting practices alone.

### Strengths and limitations

A key strength of this study is that it is among the first to examine the associations between parental attitudes, gambling behavior, and the moderating role of resilience in a convenience sample of Turkish adults residing in İstanbul. The use of validated measurement instruments and the simultaneous evaluation of both maternal and paternal parenting attitudes provide a comprehensive understanding of familial influences on gambling behavior. Although the findings contribute to the limited literature on gambling behavior in Türkiye, they should be interpreted within the context of the study sample and should not be considered nationally representative. Furthermore, the study offers culturally contextualized evidence regarding gambling behavior in Türkiye, where empirical research on this topic remains limited.

Several limitations should also be acknowledged. First, the cross-sectional design precludes causal inferences regarding the relationships among parental attitudes, resilience, and gambling behavior. Second, all variables were assessed using self-report measures. Given that gambling is socially stigmatized in Türkiye, participants may have underreported gambling-related behaviors because of social desirability bias, which may partly explain the relatively low SOGS scores observed in this study. Third, participants were recruited through convenience sampling and were limited to adults residing in İstanbul. Therefore, the findings cannot be assumed to represent the broader Turkish adult population and should be interpreted within the context of the study sample. Furthermore, demographic covariates (e.g., age, gender, and income) were not included in the moderation models. This decision was made to preserve model stability given the limited number of non-zero gambling cases and the markedly zero-inflated distribution of the outcome variable. Consequently, the reported associations should be interpreted without adjustment for these potential confounding variables. Finally, the SOGS outcome exhibited a markedly zero-inflated and positively skewed distribution, with 75.1% of participants obtaining a score of zero. This limited variability in the outcome may have reduced the statistical power to detect stronger associations and interaction effects. Although the additional Spearman correlation analyses yielded findings that were highly consistent with the Pearson correlations, the distributional characteristics of the outcome variable may have affected the stability of the OLS-based moderation estimates. Therefore, the moderation findings should be interpreted with appropriate caution. In addition, because the moderation analyses were conducted using the MedMod module in Jamovi, overall model-fit statistics (e.g., *R*^2^, Δ*R*^2^, and overall *F* statistics) and interaction-specific effect-size indices were not available for reporting. Consequently, the magnitude of the moderation models could not be evaluated using these indices and was instead interpreted based on the unstandardized interaction coefficients, their confidence intervals, significance tests, and conditional effects.

### Future directions

Future research should recruit larger, geographically diverse, and more representative samples from different regions of Türkiye to improve the generalizability of the findings and provide more accurate estimates of gambling prevalence. Longitudinal studies are also recommended to clarify the temporal and potentially causal relationships among parental attitudes, resilience, and gambling behavior. Moreover, future research should continue examining resilience as a moderator while incorporating broader individual, familial, social, and cultural factors that may contribute to gambling behavior. In addition, future studies should consider adjusting for important demographic variables, such as age, gender, and income, to determine whether the observed associations remain robust after controlling for these potential confounding factors. Given the highly zero-inflated distribution of gambling severity observed in the present study, future studies should consider applying statistical approaches specifically designed for zero-inflated or count outcomes (e.g., Poisson, negative binomial, zero-inflated, or Tobit models) to evaluate the robustness of these findings. Studies including larger samples of individuals who actively engage in gambling would also facilitate more reliable moderation analyses and provide further evidence regarding the protective role of resilience. Finally, intervention studies evaluating resilience-enhancing and family-based prevention programs would provide valuable evidence for reducing gambling-related harm.

### Practical implications

The findings of the present study may have several implications for psychiatric nursing practice; however, these implications should be interpreted in light of the characteristics of the study sample. Psychiatric nurses should routinely assess gambling behaviors together with important psychosocial risk and protective factors, including perceived parental attitudes and resilience, to facilitate the early identification of individuals at risk. Assessment of family dynamics may help identify individuals who are particularly vulnerable because of perceived parental rejection or overprotective parenting.

Resilience-enhancing interventions, psychoeducation, and counseling programs may strengthen adaptive coping strategies and reduce vulnerability to gambling-related behaviors. Family-centered interventions that promote emotional warmth, healthy parent–child communication, and supportive family relationships may further strengthen resilience and contribute to the prevention of gambling-related harm.

Psychiatric nurses can also provide psychoeducation aimed at reducing stigma surrounding gambling problems, facilitating safe self-expression, and encouraging timely help-seeking. Finally, nursing education and continuing professional development should strengthen competencies related to behavioral addictions, family assessment, resilience-based interventions, and culturally sensitive prevention strategies.

## Conclusion

This study is among the first to examine the moderating role of resilience in the relationship between perceived parental attitudes and gambling behavior among a convenience sample of adults residing in İstanbul, Türkiye. Consistent with the general objective of the study, the findings demonstrated that perceived parental attitudes and resilience were both associated with gambling behavior, although not all hypothesized relationships were supported. Specifically, hypotheses H1a and H1b were supported, indicating that higher levels of perceived maternal and paternal rejection were associated with greater gambling severity, whereas the remaining parental attitude hypotheses were not confirmed. In line with hypotheses H2a and H2b, resilience significantly moderated the associations between both maternal and paternal rejection and gambling behavior, suggesting that resilience may function as an important protective factor by buffering the adverse associations of perceived parental rejection. However, the remaining moderation hypotheses were not supported, indicating that the protective role of resilience may be specific to parental rejection rather than to all parental attitude dimensions. These findings contribute to the emerging literature on gambling behavior by improving our understanding of the interplay between familial and individual factors within the context of this study sample and highlight resilience as a promising target for future prevention and intervention research.

## Data Availability

The dataset(s) supporting the conclusions of this article is (are) available and will be provided by the authors upon request. Requests to access the datasets should be directed to Tuğba Pehlivan Sarıbudak, tugba.pehlivansaribudak@gmail.com.
